# Intrathecal Onasemnogene Abeparvovec for Sitting, Nonambulatory Patients with Spinal Muscular Atrophy: Phase I Ascending-Dose Study (STRONG)

**DOI:** 10.3233/JND-221560

**Published:** 2023-05-02

**Authors:** Richard S. Finkel, Basil T. Darras, Jerry R. Mendell, John W. Day, Nancy L. Kuntz, Anne M. Connolly, Craig M. Zaidman, Thomas O. Crawford, Russell J. Butterfield, Perry B. Shieh, Gihan Tennekoon, John F. Brandsema, Susan T. Iannaccone, John Shoffner, Sarah Kavanagh, Thomas A. Macek, Sitra Tauscher-Wisniewski

**Affiliations:** a Center for Experimental Neurotherapeutics, St. Jude Children’s Research Hospital, Memphis, TN, USA; b Division of Neurology, Nemours Children’s Hospital, Orlando, FL, USA; c Department of Neurology, Boston Children’s Hospital, Boston, MA, USA; d Center for Gene Therapy, Nationwide Children’s Hospital, Columbus, OH, USA; e Department of Pediatrics and Department of Neurology, The Ohio State University,Columbus, OH, USA; f Department of Neurology, Stanford University Medical Center, Stanford, CA, USA; g Division of Neurology, Ann and Robert H. Lurie Children’s Hospital of Chicago, Chicago, IL, USA; h Division of Neurology, Nationwide Children’s Hospital, Columbus, OH, USA; i Department of Neurology, Division of Pediatric Neurology,Washington University School of Medicine, St. Louis, MO, USA; j Department of Neurology, Johns Hopkins University School of Medicine, Baltimore, MD, USA; k Department of Pediatrics, University of Utah Health, Salt Lake City, UT, USA; l Department of Neurology, David Geffen School of Medicine at UCLA, Los Angeles, CA, USA; m Division of Neurology, Children’s Hospital of Philadelphia, Philadelphia, PA, USA; n Department of Pediatrics, University of Texas Southwestern Medical Center, Dallas, TX, USA; o Novartis Gene Therapies, Inc., Bannockburn, IL, USA; p Sangamo Therapeutics, Inc., Richmond, CA, USA

**Keywords:** Adeno-associated virus, clinical trial, gene therapy, Hammersmith Functional Motor Scale Expanded, intrathecal administration, motor milestones, neurodegenerative disorders, onasemnogene abeparvovec, spinal muscular atrophy, vector genomes

## Abstract

**Background:**

Spinal muscular atrophy (SMA) is a neuromuscular disorder arising from biallelic non-functional *survival motor neuron 1* (*SMN1*) genes with variable copies of partially functional *SMN2* gene. Intrathecal onasemnogene abeparvovec administration, at fixed, low doses, may enable treatment of heavier patients ineligible for weight-based intravenous dosing.

**Objective:**

STRONG (NCT03381729) assessed the safety/tolerability and efficacy of intrathecal onasemnogene abeparvovec for sitting, nonambulatory SMA patients.

**Methods:**

Sitting, nonambulatory SMA patients (biallelic *SMN1* loss, three *SMN2* copies, aged 6–<60 months) received a single dose of intrathecal onasemnogene abeparvovec. Patients were enrolled sequentially into one of three (low, medium, and high) dose cohorts and stratified into two groups by age at dosing: younger (6–<24 months) and older (24–<60 months). Primary endpoints included safety/tolerability, independent standing ≥3 seconds (younger group), and change in Hammersmith Functional Motor Scale Expanded (HFMSE) from baseline (older group) compared with historic controls.

**Results:**

Thirty-two patients were enrolled and completed the study (medium dose, *n* = 25). All patients had one or more treatment-emergent adverse events, with one serious and related to treatment (transaminase elevations). No deaths were reported. One of 13 patients (7.7%) in the younger group treated with the medium dose achieved independent standing. At Month 12 for the older group receiving the medium dose, change from baseline in HFMSE was significantly improved compared with the SMA historic control population (*P* < 0.01).

**Conclusions:**

Intrathecal onasemnogene abeparvovec was safe and well-tolerated. Older patients treated with the medium dose demonstrated increases in HFMSE score greater than commonly observed in natural history.

## INTRODUCTION

Spinal muscular atrophy (SMA) is a progressive, monogenic neuromuscular disorder caused by loss or disabling mutation of the *survival motor neuron 1* (*SMN1*) gene that results in reduced amounts of SMN protein and motor neuron dysfunction [1]. SMA manifests across a range of clinical subtypes defined historically by maximal motor function, and severity largely correlates negatively with the number of copies of *SMN2*, a partially functional paralog of *SMN1* [2]. SMA patients with greater *SMN2* copy numbers tend to have milder disease courses [3, 4]. The majority of patients with three *SMN2* copies will be able to achieve sitting, but not walking independently (i.e., SMA type 2) [5, 6], with progressive weakness typical of all types of SMA. Milder forms of SMA progress more slowly than types with earlier, more severe manifestation, and those infants who are able to sit are at risk for joint contractures, scoliosis, dysphagia, and respiratory complications, as well as loss of independent sitting [6–8].

US Food and Drug Administration (FDA)–approved SMA therapies include nusinersen and risdiplam; both target improving the function of the paralog *SMN2* gene [9, 10]. In contrast, onasemnogene abeparvovec is a gene replacement therapy that delivers the *SMN* transgene via an adeno-associated virus serotype 9 (AAV9) vector that persists as an episome in postmitotic tissues [11]. Onasemnogene abeparvovec is broadly distributed to tissues following intravenous administration [12, 13]. Intrathecal delivery also results in broad systemic distribution to other tissues [13]. Onasemnogene abeparvovec is a weight-based therapy with a recommended dosage of 1.1×10^14^ vector genomes (vg) per kg of body weight.

Intravenous onasemnogene abeparvovec efficacy and safety were demonstrated in one Phase I and two Phase III studies that included symptomatic SMA type 1 patients with two *SMN2* copies [14–17], and a Phase III study that included presymptomatic infants at risk of developing SMA with two or three *SMN2* copies [18, 19]. In symptomatic patients, intravenous onasemnogene abeparvovec improved survival, motor function, and motor milestone achievement over natural history and decreased nutritional and respiratory support requirements [14–17]. Administration in presymptomatic children with biallelic *SMN1* mutations treated at ≤6 weeks of life led to further improvements, with many motor milestones achieved within normal developmental windows [18, 19].

A favorable benefit-risk profile has been demonstrated for intravenous onasemnogene abeparvovec for symptomatic SMA type 1 patients, with increases in liver transaminases and decreases in platelets being the most commonly observed adverse events (AEs) [14–17]. Safety findings from preclinical studies, clinical studies, and post-marketing data identified the following AEs of special interest (AESIs): hepatotoxicity, thrombocytopenia, thrombotic microangiopathy (TMA), cardiac AEs, and ganglionopathy [20]. No treatment-related, serious AEs (SAEs) were observed with presymptomatic administration [18, 19].

SMA is diagnosed in an increasing number of jurisdictions by newborn screening protocols, but those affected children with milder forms will not be identified until onset of symptoms beyond the window of approved intravenous onasemnogene abeparvovec treatment. The necessary greater doses of onasemnogene abeparvovec that would be dictated by weight-based dosing in this group raise further safety considerations because of the greater viral load. In contrast, intrathecal administration could enable a fixed-dose administration of onasemnogene abeparvovec directly into the intrathecal space of the central nervous system (CNS) and allow for greater neuronal transduction on a vg/kg basis. In preclinical studies, delivery directly into the cerebrospinal fluid (CSF) via intrathecal injection reduced the amount of viral vector administered by a factor of nearly 10, with equal distribution and efficacy throughout the CNS and reduced viral vector loads in major peripheral organs (e.g., liver) [13, 21–23]. In nonhuman primates (NHPs) administered self-complementary adeno-associated virus serotype 9–chicken *β*-actin promoter–green fluorescent protein (sc-AAV9-CB-GFP) intrathecally via lumbar puncture or the intracisterna magna (1.0×10^13^ or 3.0×10^13^ vg/animal), widespread biodistribution was observed in the spinal cord (spinal cord lower motor neurons), dorsal root ganglia (DRG), and liver [13]. As such, intrathecal administration of onasemnogene abeparvovec could address a significant unmet medical need in the treatment of heavier and adult patients with SMA with potential for improving motor function and overall quality of life.

The objective of STRONG (NCT03381729), a Phase I, open-label, ascending-dose trial, was to assess the safety, tolerability, and efficacy of intrathecal onasemnogene abeparvovec for sitting, nonambulatory patients with SMA.

## MATERIALS AND METHODS

### Study design

STRONG was a Phase I, open-label, ascending-dose study conducted at 11 centers in the United States. The study was conducted in accordance with the Declaration of Helsinki, International Council for Harmonization/Good Clinical Practice guidelines, and applicable regulatory requirements (e.g., those relating to informed consent and the protection of human patients in biomedical research). The study was approved by institutional review boards at all participating institutions, and written informed consent was obtained from parents or legal guardians of enrolled patients.

### Patients

**Fig. 1:**
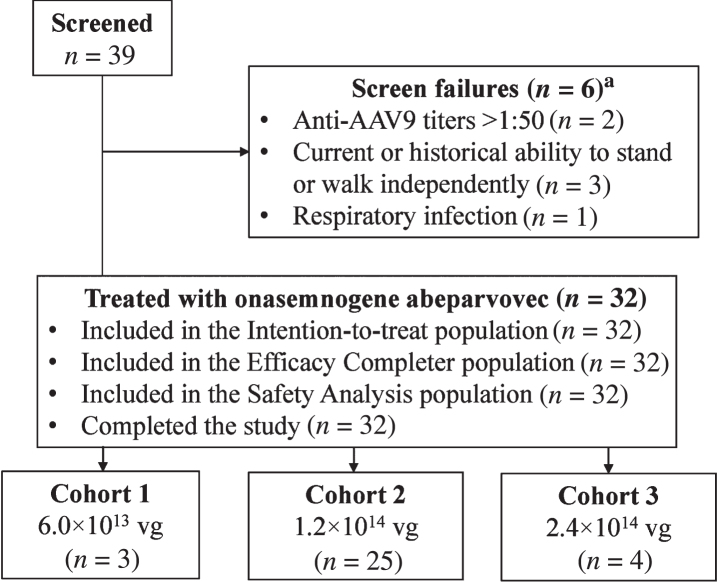
Patient disposition. AAV9, adeno-associated virus-9; vg, vector genomes. ^a^One of the six patients was rescreened under two different patient identifications and failed screening both times.

Participants had genetic confirmation of SMA (biallelic deletion of *SMN1*) and three copies of *SMN2* without the genetic modifier (c.859G>C) [24]. All were able to sit unassisted for 10 or more seconds but could not stand or walk independently at the time of study entry or at any prior time point. Patients were enrolled sequentially into one of three dose cohorts: low, 6.0×10^13^ vg; medium, 1.2×10^14^ vg; and high, 2.4×10^14^ vg (Fig. 1). Patients were stratified into two groups within each cohort based on age at dosing: a younger group 6 to <24 months of age and an older group 24 to <60 months of age. The study planned to enroll at least 27 (up to 51, if the high dose was tested) patients, including at least 15 patients in the younger group and 12 patients in the older group. Enrollment was terminated early with four patients in the high-dose cohort because of a partial clinical hold from the FDA on the intrathecal program. Full eligibility criteria are described in the Supplementary Appendix. All patients in the low- and high-dose cohorts were in the younger group.

### Procedures

Patients received oral prophylactic prednisolone (approximately 1 mg/kg/day) 24 hours prior to intrathecal onasemnogene abeparvovec dosing. Prophylactic treatment continued for approximately 56 days in accordance with the following treatment guidance: 1 mg/kg/day until at least 30 days post injection, 0.5 mg/kg/day at Weeks 5 and 6 post injection, 0.25 mg/kg/day at Weeks 7 and 8 post injection, and discontinuation at Week 9 if liver testing results were unremarkable.

Onasemnogene abeparvovec was delivered as a single intrathecal injection under sterile conditions with fluoroscopic/radiographic guidance per institutional guidelines. Sedation/anesthesia was required for all patients. Following administration of vector, to enhance distribution to cervical and brain regions, patients were placed in the Trendelenburg position, tilted head down at 30° for 15 minutes [23]. Patients were observed at the hospital for 48 hours post intrathecal injection. Patients enrolled in the low- and medium-dose cohorts completed 12 months of follow-up post intrathecal administration. Patients enrolled in the high-dose cohort completed 15 months of follow-up. When patients completed the study, they were invited to participate in a long-term follow-up study conducted under a separate protocol.

### Safety analysis

Safety was assessed as a primary endpoint by monitoring AE reports and concomitant medication usage, and evaluating physical examinations, vital signs, cardiovascular evaluations, and laboratory results. Five categories of potential AESIs included hepatotoxicity, thrombocytopenia, cardiac events, TMA, and clinical manifestations consistent with sensory ganglionopathy [20]. To systematically search for these AESIs, specific standardized or customized Medical Dictionary for Regulatory Activities (MedDRA^®^) queries related to each category were defined (see Supplementary Methods for additional information). Because of overlap in the search strategies, certain events (e.g., hepatomegaly) appear in more than one AESI category. The secondary safety endpoint was the average number of hours per day of noninvasive ventilatory support.

### Efficacy outcomes

Efficacy and safety of intrathecal onasemnogene abeparvovec administration were assessed independently for each age group. For patients in the younger group, the prespecified primary efficacy endpoint was the percentage of patients who achieved the ability to stand without support for at least 3 seconds (Bayley Scales of Infant and Toddler Development, Version 3 [Bayley-III] item #40), which was video-confirmed and centrally reviewed. For patients in the older group, the prespecified primary efficacy endpoint was the change from baseline in Hammersmith Functional Motor Scale Expanded (HFMSE) [25], a validated SMA-specific assessment devised for evaluating motor ability and clinical progression. The HFMSE contains 33 items rated from 0 (unable to perform) to 2 (performs without modification/adaptation/compensation), and total scores range from 0–66, with greater scores indicative of greater degrees of motor ability. The prespecified secondary efficacy endpoint for both age groups was the percentage of patients who achieved the ability to walk without assistance (video-confirmed and centrally reviewed), defined as taking at least five steps independently, displaying coordination and balance (Bayley-III item #43).

Exploratory endpoints included achievement of motor milestones, which were also captured during videotaping sessions during site visits and/or provided by a parent or legal guardian for all patients in both age groups. Other exploratory endpoints for patients in the younger group included change from baseline in fine and gross motor components of the Bayley-III and change in HFMSE for patients who continued in the study past 24 months of age and had at least 6 months of HFMSE data. The score obtained at the first assessment when patients reached 24 months of age was used as the baseline score. Change from baseline in fine and gross motor components of the Bayley-III was an exploratory endpoint for patients in the older group.

### Statistical analysis

The primary and secondary analyses were based on the intention-to-treat (ITT) set and compared patients treated with the medium dose of onasemnogene abeparvovec with a population-matched cohort from the Pediatric Neuromuscular Clinical Research (PNCR) natural history data set [26] as a prespecified analysis (Protocol Amendment 3, 17 February 2017). Efficacy analysis was not undertaken for the low- and high-dose cohorts as they were not adequately powered to perform the analysis.

For purposes of this study, sitting (for SMA type 2) was defined as being able to sit independently for >10 seconds (World Health Organization Multicentre Growth Reference Study [WHO-MGRS] criteria) at study entry. In addition, standing was defined per Bayley-III item #40 for onasemnogene abeparvovec–treated patients. This milestone was defined as a score of 2 on item #19 of the HFMSE at any time for the PNCR cohort. Walking independently was defined per Bayley-III item #43 for onasemnogene abeparvovec–treated patients and by achieving a score of 2 points on item #20 of the HFMSE at any time for the PNCR cohort. All motor milestones achieved by onasemnogene abeparvovec–treated patients were video-confirmed and centrally reviewed.

All patients with SMA types 2 or 3 who provided sufficient records and follow-up for evaluation and met entry criteria for STRONG (three copies of *SMN2*, symptom onset before 12 months of age, and baseline and follow-up visits within the age criteria for the study) were considered for inclusion in the natural history comparison data set. For comparison with patients in the younger group, the PNCR natural history population encompassed all 51 patients enrolled in the PNCR study who had SMA types 2 or 3, three copies of *SMN2*, symptom onset before 12 months of age, and at least one visit at or before 36 months of age. The primary PNCR population used comparison of change from baseline in HFMSE scores of patients in the older group. This population was a subset of 15 patients drawn from the PNCR cohort who had SMA types 2 or 3, three copies of *SMN2*, symptom onset before 12 months of age, diagnosis before 24 months of age, were unable to stand or walk at enrollment (baseline visit), received an HFMSE evaluation between 24 and 60 months of age (“baseline”), and had a follow-up evaluation (Hammersmith Functional Motor Scale [HFMS]) of HFMSE performed between 12 and 14 months following that baseline evaluation. The *SMN2* modifier variant (c.859G>C) [24] was not assessed in the PNCR study cohort.

The percentage achieving the ability to stand without support up to the 12-month study visit was compared between younger patients treated with intrathecal onasemnogene abeparvovec (medium dose, ITT population, *n* = 13) and the PNCR natural history control population (*n* = 51). Based upon a review of eligibility-matched patients from the PNCR, 14% of PNCR patients who met the study criteria and were 6 to <24 months of age achieved the ability to stand without support, defined as achieving a score of 2 on item #19 of the HFMSE, and 10% achieved the ability to walk without assistance, defined as achieving a score of 2 on item #20 of the HFMSE. We expected 85% of treated patients in the younger group to achieve the ability to stand alone and 60% to achieve the ability to walk without assistance. A sample size of 12 patients would provide power of >90% to detect a significant difference compared with the PNCR natural history control population with *α*= 0.05 using a two-sample, two-sided superiority Fisher exact test. The difference in percentage of patients achieving each milestone, 95% CIs for the difference in percentages, and *P*-values were reported for the medium-dose cohort.

For patients in the older group treated with the medium dose (ITT population, *n* = 12), the change from baseline in HFMSE was analyzed using a mixed model with repeated measurement. Based on a review of eligibility-matched patients from the primary PNCR population, a mean change of –1.33 points (SD, 4.32 points) was observed at 12 months from baseline for PNCR patients aged 2 to 5 years with three copies of *SMN2*. The power calculation was based on the assumption of a mean increase of eight points from baseline on the HFMSE with equivalent variance. Based on these assumptions, 12 patients in the older group would have >90% power to detect a significant difference with *α*= 0.05 when compared with patient-level data available from the primary PNCR population. The unadjusted means, least squares (LS) means, differences between LS means, 95% two-sided CIs for each difference, and the *P*-values from model effects were reported for each scheduled visit for the medium-dose cohort.

The population used for safety analyses was the safety analysis set (*n* = 32). Safety was assessed on the basis of AEs, clinical laboratory data, physical examinations, noninvasive ventilatory support use, vital signs, and related examinations. All safety analyses were summarized overall and by actual dose received and age group.

## RESULTS

### Patient disposition

Thirty-nine patients were screened, and 32 patients were enrolled: low-dose cohort, *n* = 3; medium-dose cohort, *n* = 25 (younger group, *n* = 13 and older group, *n* = 12); and high-dose cohort, *n* = 4 (Fig. 1). The first patient enrolled on December 21, 2017, and the last patient completed the last visit on May 10, 2021. After enrollment into the low- and medium-dose cohorts was completed, and enrollment into the high-dose cohort was ongoing, the FDA imposed a partial clinical hold on the intrathecal clinical program because of safety concerns that emerged from an experimental study with non-GLP material that revealed DRG neuropathologic findings in NHPs without apparent clinical features [27]. Thus, enrollment into the study was suspended, with four patients (of 24 planned) enrolled into the high-dose cohort. After the results of further long-term nonclinical safety studies were communicated to the FDA, including nonprogressive microscopic DRG findings that were not associated with detectable electrophysiology changes [28], the partial clinical hold was lifted on July 30, 2021. During this period, the AE profile was searched for signs or symptoms of DRG toxicity (e.g., transient pain or muscle weakness following administration). No subacute or chronic findings that would implicate DRG toxicity were identified. The sponsor determined that this study had met its overall strategic objectives within the broader intrathecal clinical development program, and the decision was made not to enroll additional patients into the high-dose cohort, with the medium dose providing the optimal benefit-risk profile for patients. Enrollment was terminated early, and the study was declared complete on November 18, 2021. All enrolled patients completed the study.

### Demographics and baseline clinical characteristics

Key demographics and baseline characteristics for both the safety analysis set and the PNCR-matched control populations are summarized in Table 1. The median age at intrathecal onasemnogene abeparvovec administration was 20.3 months (range, 7–55). No patient required non-oral feeding or ventilator support at baseline.

**Table 1:** Demographics and baseline clinical characteristics (safety population)

	STRONG					PNCR	
	Low dose 6.0×10^13^ vg (*n* = 3)	Medium dose 1.2×10^14^ vg (*n* = 25)		High dose 2.4×10^14^ vg (*n* = 4)	All patients (N = 32)	PNCR natural history control population^a^	Primary PNCR population^b^
	Younger group	Younger group	Older group	Younger group		Comparison group for age <24 months (*n* = 51)	Comparison group for >24 months and ≤60 months (*n* = 15)
	6 to <24 months	6 to <24 months (*n* = 13)	24 to <60 months (*n* = 12)	6 to <24 months
Age at baseline or PNCR entry, months							
Median (range)	18.9 (13–20)	17.7 (7–23)	33.7 (26–55)	17.4 (10–23)	20.3 (7–55)	67.5 (11–390)^c^	43.1 (29–56)^c^
Age at symptom onset, median (range), months	9.0 (7–11)	8.0 (0–10)^d^	8.5 (5–11)	8.0 (1–9)^d^	8.0 (0–11)	N/A	N/A
Age (months) at first HFMSE assessment conducted after reaching 24 months of age^e^							43.1 (29–56)
Sex, *n* (%)
Male	1 (33.3)	7 (53.8)	6 (50.0)	4 (100)	18 (56.3)	25 (49.0)	9 (60.0)
Female	2 (66.7)	6 (46.2)	6 (50.0)	0	14 (43.8)	26 (51.0)	6 (40.0)
Race, *n* (%)
White	2 (66.7)	10 (76.9)	8 (66.7)	3 (75.0)	23 (71.9)	31 (60.8)	9 (60.0)
Asian	0	1 (7.7)	4 (33.3)	1 (25.0)	6 (18.8)	6 (11.8)	1 (6.7)
Other	0	1 (7.7)	0	0	1 (3.1)
Multiple	1 (33.3)	1 (7.7)	0	0	2 (6.3)	6 (11.8)	0
Missing	0	0	0	0	0	8 (15.7)	5 (33.3)
Ethnicity, *n* (%)
Hispanic or Latino	2 (66.7)	3 (23.1)	0	0	5 (15.6)	6 (11.8)	2 (13.3)
Not Hispanic or Latino	1 (33.3)	10 (76.9)	12 (100)	4 (100)	27 (84.4)	38 (74.5)	9 (60.0)
Missing	0	0	0	0	0	7 (13.7)	4 (26.7)
Baseline weight, kg
Median (range)	9.9 (8.0–11.8)	9.5 (8.3–10.8)	12.7 (9.8–20.2)	9.1 (8.7–9.5)	10.0 (8.0–20.2)	16.0 (8–54), *n* = 43	12.5 (11–15), *n* = 10
Baseline length/height, cm
Median (range)	74.9 (73–82)	75.5 (69–87)	89.0 (83–112)	73.3 (71–77)	80.3 (69–112)	114.0 (73–159), *n* = 43	94.0 (89–106), *n* = 11
Feeding support, *n* (%)
Yes	0	0	0	0	0
No	3 (100)	13 (100)	12 (100)	4 (100)	32 (100)
Ventilatory support, *n* (%)
Yes	0	0	1 (8.3)^f^	0	1 (3.1)
No	3 (100)	13 (100)	11 (91.7)	4 (100)	31 (96.9)
Gestational age at birth, weeks
Median (range)	39 (37–39)	39 (38–41)	40 (35–42)^g^	40 (39–41)	39 (35–42)	40.0 (32–43), *n* = 44	40.0 (36–41), *n* = 15
HFMSE score,^h^ median (range)	–	–	12.0 (3–32)	–	–
Bayley-III raw gross motor score, median (range)	28.0 (17–34)	20.0 (14–30)	20.0 (16–35)	22.0 (18–32)	–
Bayley-III raw fine motor score, median (range)	33.0 (28–33)	31.0 (22–38)	47 (32–60)	32.5 (22–43)	–

HFMSE, Hammersmith Functional Motor Scale Expanded; N/A, not assessed; PNCR, Pediatric Neuromuscular Clinical Research; SMA, spinal muscular atrophy; vg, vector genomes. Younger group, 6 to <24 months of age at dosing; older group, 24 to <60 months of age at dosing. ^a^Includes all patients enrolled in the PNCR study who met the criteria of having SMA types 2 or 3, three copies of *SMN2*, symptom onset before 12 months of age, and at least one visit at or before 36 months of age. ^b^Contains a subset of 15 patients from the PNCR natural history control population who had SMA types 2 or 3, three copies of *SMN2*, symptom onset before 12 months of age, diagnosis before 24 months of age, were unable to stand or walk at enrollment (baseline visit), received an HFMSE evaluation between 24 and 60 months of age (“baseline”), and had a follow-up evaluation (HFMS of HFMSE performed between 12 and 14 months following that baseline evaluation. ^c^Age = (date of PNCR entry –date of birth + 1)/30, rounded to first decimal. ^d^Patient 11 had SMA symptom onset at 0 months of age with one SMA symptom reported at baseline: “Genetically positive for SMA.” Patient 29 had SMA symptom onset at 1 month of age, with the following SMA symptoms reported at baseline: constipation, developmental delay, hypotonia, limb weakness, poor weight gain, and sweating with sleep. ^e^Age = (date of earliest HFMSE assessment conducted after reaching 24 months of age –date of birth +1/30), rounded to first decimal. This was only calculated for the primary PNCR comparator group. ^f^Patient 26 reported noninvasive ventilatory support on date of birth and 1 day after birth. ^g^*n* = 11. ^h^HFMSE is a scale used to investigate a child’s ability to perform various activities and is used in later-onset (type 2 or type 3) SMA. The scale has 33 items scored as 0, 1, or 2 (maximum score = 66, with a higher score representing a greater level of function). HFMSE is intended for individuals older than 24 months of age.

All patients received the entire volume of onasemnogene abeparvovec. Overall, prednisolone was administered for a median of 60.5 days (range, 3–75 days) with a dosage that ranged from 1 mg/kg/day to 0.25 mg/kg/day over the initiation and tapering periods, respectively. One patient received prednisolone for 3 days, after which they were given oral prednisone from Day 2 to Day 66.

### Safety results

No deaths were reported in the study. All patients had at least one treatment-emergent adverse event (TEAE) (Tables 2 and 3), most of which were Grade 1 or Grade 2. Grade 3 events were reported in nine patients (low-dose cohort, *n* = 1 and medium-dose cohort, *n* = 8) (Table 4). The most frequent events (>20% of patients) were upper respiratory tract infection (62.5%), pyrexia (56.3%), cough (34.4%), vomiting (31.3%), and constipation (21.9%) (Table 3). SAEs were reported in seven patients (21.9%) (low-dose cohort, *n* = 1 and medium-dose cohort, *n* = 6) (Supplementary Table 1). Of the 12 TEAEs considered related to study treatment by the investigator (medium-dose cohort, *n* = 11, and high-dose cohort, *n* = 1) (Supplementary Table 2), one was serious (Patient 14 in the medium-dose cohort reported elevated alanine aminotransferase [ALT; Grade 3] and aspartate aminotransferase [AST; Grade 2]).

**Table 2:** Overview of treatment-emergent adverse events (safety population)

Patients, *n* (%)	Low dose 6.0×10^13^ vg (*n* = 3)	Medium dose 1.2×10^14^ vg (*n* = 25)		High dose 2.4×10^14^ vg (*n* = 4)	Overall (N = 32)
	Younger group	Younger group (*n* = 13)	Older group (*n* = 12)	Younger group	All ages
Any TEAE	3 (100)	13 (100)	12 (100)	4 (100)	32 (100)
Grade ≥3 TEAE	1 (33.3)	4 (30.8)	4 (33.3)	0	9 (28.1)
Treatment-related TEAE^a^	0	7 (53.8)	4 (33.3)	1 (25.0)	12 (37.5)
Serious TEAEs	1 (33.3)	2 (15.4)	4 (33.3)	0	7 (21.9)
Serious TEAEs related to study treatment	0	1 (7.7)	0	0	1 (3.1)
TEAEs resulting in death	0	0	0	0	0

TEAE, treatment-emergent adverse event; vg, vector genomes. Younger group, 6 to <24 months of age at dosing; older group, 24 to <60 months of age at dosing. ^a^Adverse events were considered related to treatment if the event was classified as possibly, probably, or definitely related to study treatment by the study investigator.

**Table 3:** Summary of TEAEs in two or more patients overall by preferred term (safety population)

Preferred term	Low dose (6.0×10^13^ vg; *n* = 3)	Medium dose (1.2×10^14^ vg; *n* = 25)		High dose (2.4×10^14^ vg; *n* = 4)	Overall (N = 32)
	Younger group,	Younger group,	Older group,	Younger group,	All ages,*n* (%)
	*n* (%)	*n* (%)	*n* (%)	*n* (%)
**Any TEAE**	3 (100)	13 (100)	12 (100)	4 (100)	32 (100)
Upper respiratory tract infection	2 (66.7)	10 (76.9)	5 (41.7)	3 (75.0)	20 (62.5)
Pyrexia	3 (100)	6 (46.2)	7 (58.3)	2 (50.0)	18 (56.3)
Cough	0	3 (23.1)	7 (58.3)	1 (25.0)	11 (34.4)
Vomiting	0	5 (38.5)	3 (25.0)	2 (50.0)	10 (31.3)
Constipation	0	2 (15.4)	3 (25.0)	2 (50.0)	7 (21.9)
Nasopharyngitis	0	3 (23.1)	2 (16.7)	1 (25.0)	6 (18.8)
Rash	1 (33.3)	2 (15.4)	3 (25.0)	0	6 (18.8)
Blood alkaline phosphatase increased	1 (33.3)	3 (23.1)	0	1 (25.0)	5 (15.6)
Nasal congestion	1 (33.3)	2 (15.4)	2 (16.7)	0	5 (15.6)
Rhinorrhea	0	2 (15.4)	2 (16.7)	1 (25.0)	5 (15.6)
Dermatitis diaper	1 (33.3)	2 (15.4)	0	1 (25.0)	4 (12.5)
Otitis media	1 (33.3)	0	3 (25.0)	0	4 (12.5)
Scoliosis	0	0	4 (33.3)	0	4 (12.5)
Tachycardia	0	2 (15.4)	2 (16.7)	0	4 (12.5)
Teething	1 (33.3)	2 (15.4)	0	1 (25.0)	4 (12.5)
Arthropod bite	0	3 (23.1)	0	0	3 (9.4)
Hypertension	0	3 (23.1)	0	0	3 (9.4)
Lymphadenopathy	0	2 (15.4)	1 (8.3)	0	3 (9.4)
Pneumonia	1 (33.3)	0	2 (16.7)	0	3 (9.4)
Upper respiratory tract congestion	0	2 (15.4)	1 (8.3)	0	3 (9.4)
Viral infection	0	1 (7.7)	1 (8.3)	1 (25.0)	3 (9.4)
Weight gain poor	0	3 (23.1)	0	0	3 (9.4)
Aspartate aminotransferase increased	0	1 (7.7)	1 (8.3)	0	2 (6.3)
Conjunctivitis	0	2 (15.4)	0	0	2 (6.3)
Contusion	0	2 (15.4)	0	0	2 (6.3)
Dehydration	0	0	2 (16.7)	0	2 (6.3)
Ear infection	0	1 (7.7)	1 (8.3)	0	2 (6.3)
Eczema	0	1 (7.7)	1 (8.3)	0	2 (6.3)
Erythema	0	1 (7.7)	1 (8.3)	0	2 (6.3)
Hypotension	1 (33.3)	0	1 (8.3)	0	2 (6.3)
Joint contracture	0	0	2 (16.7)	0	2 (6.3)
Kyphosis	0	2 (15.4)	0	0	2 (6.3)
Limb asymmetry	1 (33.3)	1 (7.7)	0	0	2 (6.3)
Mitral valve incompetence	0	1 (7.7)	1 (8.3)	0	2 (6.3)
Pain in extremity	0	0	2 (16.7)	0	2 (6.3)
Respiration abnormal	0	1 (7.7)	1 (8.3)	0	2 (6.3)
Respiratory tract infection viral	0	0	2 (16.7)	0	2 (6.3)
Rhinovirus infection	0	0	2 (16.7)	0	2 (6.3)
Seasonal allergy	0	0	1 (8.3)	1 (25.0)	2 (6.3)
Sinus tachycardia	0	1 (7.7)	1 (8.3)	0	2 (6.3)
Skin abrasion	0	0	2 (16.7)	0	2 (6.3)
Sleep apnea syndrome	0	1 (7.7)	1 (8.3)	0	2 (6.3)

TEAE, treatment-emergent adverse event; vg, vector genomes. Younger group, 6 to <24 months of age at dosing; older group, 24 to <60 months of age at dosing. Note: TEAEs are classified by preferred term using MedDRA^®^, Version 23.0.

**Table 4:** TEAEs by maximum severity (safety population)

	Low dose (6.0×10^13^ vg; *n* = 3)	Medium dose (1.2×10^14^ vg; *n* = 25)		High dose (2.4×10^14^ vg; *n* = 4)	Overall (N = 32)
	Younger group, *n* (%)	Younger group, *n* (%)	Older group, *n* (%)	Younger group, *n* (%)	All ages, *n* (%)
**Any TEAE**	3 (100)	13 (100)	12 (100)	4 (100)	32 (100)
Grade 1 (mild)	1 (33.3)	3 (23.1)	3 (25.0)	2 (50.0)	9 (28.1)
Grade 2 (moderate)	1 (33.3)	6 (46.2)	5 (41.7)	2 (50.0)	14 (43.8)
Grade 3 (severe)	1 (33.3)	4 (30.8)	4 (33.3)	0	9 (28.1)
Grade 4 (life-threatening)	0	0	0	0	0
Grade 5 (fatal)	0	0	0	0	0

TEAE, treatment-emergent adverse event; vg, vector genomes. Younger group, 6 to <24 months of age at dosing; older group, 24 to <60 months of age at dosing.

Five AESIs were also evaluated: hepatotoxicity, thrombocytopenia, cardiac events, TMA, and sensory abnormalities suggestive of ganglionopathy. Nine hepatotoxicity events in seven patients (21.9%) were reported (Supplementary Table 3). Five of these patients had isolated increases in blood alkaline phosphatase reported as AEs, none of which were considered related to study treatment by the investigator. The increases in alkaline phosphatase were likely related to bone in these growing children and indicative of transient childhood hyperphosphatemia, not hepatotoxicity. Two patients (Patients 14 and 20 in the medium-dose cohort) had events that were considered probably related to onasemnogene abeparvovec by the investigator, including hepatomegaly and transaminase (ALT and AST) increases in one patient and AST increased in the second patient. All hepatotoxicity events resolved. However, one event, blood alkaline phosphatase increased, was reported as resolved with sequelae (Patient 6 from the medium-dose cohort). No concomitant treatment was reported for these events with the exception of Patient 14, who received concomitant medication (prednisolone) for ALT and AST elevations as presented in Supplementary Table 3. None of the patients had concurrent elevations in ALT and/or AST >3× the upper limit of normal (ULN) and bilirubin >2× ULN, and no patients had clinical signs or symptoms associated with the elevations (i.e., jaundice).

Five thrombocytopenia events in five patients (15.6%) were reported, of which one event, prolongation of activated partial thromboplastin time, was reported for Patient 14 (medium-dose cohort) that was considered possibly related to onasemnogene abeparvovec by the investigator (Supplementary Table 4). Patient 3, who had an AE of infusion site bruising, experienced a single low platelet count of 57×10^9^/L on Study Day 2. At the next testing on Study Day 8, the platelet count had returned to within reference ranges. None of the other patients had events associated with platelet values that met the potentially clinically significant criteria of <75×10^9^/L.

Twelve cardiac events in nine patients (28.1%) in the medium-dose cohort were reported, including the event of hepatomegaly also included in the analysis of hepatotoxicity AESIs described above (Supplementary Table 5). All cardiac events were Grade 1, with the exception of a Grade 3 tachycardia in Patient 26, which resolved on the day of onset, and none were serious. Three of the 12 events were considered possibly or probably related to onasemnogene abeparvovec as assessed by the investigator, including sinus tachycardia (*n* = 1), hepatomegaly (*n* = 1), and elevation in CK-MB (*n* = 1).

No events were reported to suggest TMA or sensory ganglionopathy.

Ventilatory support requirements were evaluated as a secondary safety outcome. No patients in STRONG required invasive ventilatory support. None of the patients received bilevel positive airway pressure (BiPAP) at baseline. BiPAP use was reported for two patients in the medium-dose cohort (Patients 10 and 21) during the study. For Patient 10, the mean number of hours of BiPAP was 2.6 hours at the Month 6 visit when ventilatory support was initiated and 10.1 hours at the Month 12 visit. For Patient 21, the mean number of hours of BiPAP was 10.5 hours at the Month 2 visit and 0.04 hours at the Month 12 visit.

### Primary efficacy analysis

**Fig. 2:**
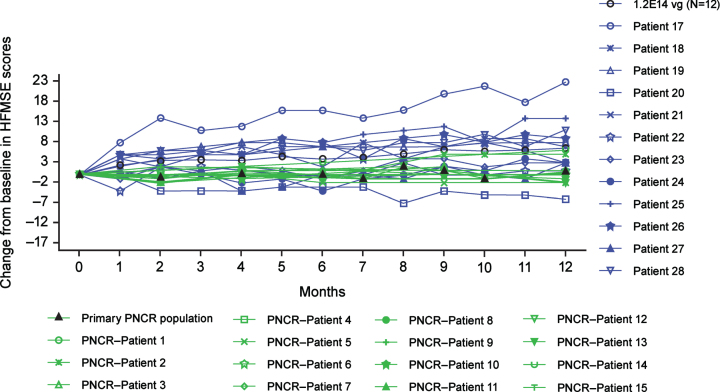
Change from baseline in HFMSE scores up to Month 12 for the older group (ITT population). All patients were treated with 1.2×10^14^ vg onasemnogene abeparvovec (medium dose) administered via intrathecal administration (N = 12). The PNCR cohort represents the primary PNCR population that contains a subset of 15 patients from the PNCR natural history control population who had SMA types 2 or 3, three copies of *SMN2*, symptom onset before 12 months of age, diagnosis before 24 months of age, were unable to stand or walk at enrollment (baseline visit), received an HFMSE evaluation between 24 and 60 months of age (“baseline”), and had a follow-up evaluation (HFMS of HFMSE performed between 12 and 14 months following that baseline evaluation. Older group, 24 to <60 months of age at dosing. HFMS, Hammersmith Functional Motor Scale; HFMSE, Hammersmith Functional Motor Scale-Expanded; ITT, intention-to-treat; PNCR, Pediatric Neuromuscular Clinical Research; vg, vector genomes.

For patients in the older group treated with the medium dose, LS mean change from baseline in HFMSE at Month 12 was 6.0 (95% CI, 3.7, 8.3) (Fig. 2; SupplementaryTable 6). In the younger group, one of 13 patients (7.7%) treated with the medium dose and one of three patients (33.3%) treated with the low dose achieved independent standing (Table 5).

**Table 5:** Patients in the younger group who achieved the ability to stand alone at any post-baseline visit up to 12 months (ITT population)

	PNCR natural	Onasemnogene abeparvovec		
	history control	Low dose 6.0×10^13^ vg (*n* = 3)	Medium dose 1.2×10^14^ vg (*n* = 13)	High dose 2.4×10^14^ vg (*n* = 4)
	population^a^ (N = 51)
Patients achieving the ability to stand alone, *n* (%)				
Yes	7 (13.7)	1 (33.3)	1 (7.7)	0
No	44 (86.3)	2 (66.7)	12 (92.3)	4 (100)
Percentage difference^b^				
Difference in percentage vs. PNCR (95% CI)			–6.0 (–21.8–22.8)
*p*-value (Fisher’s exact test)			>0.999

HFMSE, Hammersmith Functional Motor Scale Expanded; ITT, intention-to-treat; PNCR, Pediatric Neuromuscular Clinical Research; vg, vector genomes. Younger group, 6 to <24 months of age at dosing. ^a^Includes all patients enrolled in the PNCR study who met the criteria of having SMA types 2 or 3, three copies of *SMN2*, symptom onset before 12 months of age, and at least one visit at or before 36 months of age. ^b^The Fisher’s exact test was only performed for the medium dose. Note: Baseline HFMSE score for Patient 2 (low-dose group), who achieved the ability to stand alone, was 27, increasing to 35. Patient 11 (medium-dose group) did not reach 24 months of age during the study and subsequently had no HFMSE assessments.

The changes from baseline in HFMSE observed at Month 12 in the older group were significantly greater than observed in the primary PNCR population, with a LS mean difference (95% CI) of 5.5 (1.9, 9.0; *P* < 0.01). Eleven of 12 patients (91.7%) in the older group (medium dose) achieved a ≥3-point increase in HFMSE at any post-baseline visit (Supplementary Table 7), compared with two of 15 patients (13.3%) in the primary PNCR population. The estimated percentage difference for onasemnogene abeparvovec–treated patients versus the primary PNCR population was 78.3 (95% CI: 42.5, 95.0; *P* < 0.01).

No significant differences in the achievement of independent standing were observed between those younger patients treated with the medium dose (7.7%) and the PNCR natural history control population (seven of 51 patients, 13.7%), of which five had this ability at baseline (*P* > 0.999) (Table 5).

### Secondary efficacy analyses

One of 13 patients (7.7%) in the younger group treated with the medium dose walked independently for at least five steps, compared with five of 51 patients (9.8%) in the PNCR natural history control population (*P* > 0.999) (Supplementary Table 8). No older patients treated with onasemnogene abeparvovec or in the PNCR natural history control population achieved the secondary efficacy endpoint of independent walking.

### 3.6 Exploratory efficacy analyses

Bayley-III motor milestones (beyond independent sitting that all patients had achieved at baseline) achieved by all cohorts and age groups and confirmed by independent central video review are presented in Supplementary Table 9. Six patients in the younger group treated with the medium dose gained 15 motor milestones (rolls, *n* = 4, crawls, *n* = 2; pulls to stand, *n* = 2; stands with assistance, *n* = 3; stands alone, *n* = 1; walks with assistance, *n* = 2; and walks alone, *n* = 1). Three older patients in the medium-dose cohort also achieved four motor milestones (rolls, *n* = 1; stands with assistance, *n* = 2; and walks with assistance, *n* = 1).

Younger patients in the low- and high-dose cohorts also gained motor milestones beyond independent sitting. In the low-dose cohort, two patients treated at 20.2 and 18.9 months gained four motor milestones (crawls, *n* = 2; pulls to stand, *n* = 1; and stands alone, *n* = 1). In the high-dose cohort, three patients achieved three motor milestones (rolls, pulls to stand, and stands with assistance, all *n* = 1).

A second exploratory efficacy endpoint was the change from baseline in fine and gross motor components of the Bayley-III. For patients in the younger group treated with the medium dose, the median (range) maximum change from baseline in the Bayley-III gross motor subtest at any post-baseline visit up to 12 months was 5.0 (1–25), with corresponding median (range) maximum change from baseline values for the Bayley-III fine motor subtest of 12.0 (7–19) (Supplementary Table 10 and Supplementary Figure 2). For patients in the older group treated with the medium dose, the median (range) maximum change from baseline in Bayley-III at any post-baseline visit up to Month 12 was 3.0 (1–12) for the gross motor subtest and 10.5 (1–23) for the fine motor subtest (SupplementaryTable 11 and Supplementary Figure 4). Increases were also noted in younger patients treated with both low and high doses with the median (range) maximum change from baseline in the Bayley-III gross motor subtest at any post-baseline visit up to 12 months of 5.0 (5–7) in the low-dose cohort and 4.0 (2–10) in the high-dose cohort, with corresponding median (range) maximum change from baseline values for the Bayley-III fine motor subtest of 18.0 (9–19) in the low-dose cohort and 10.5 (8–19) in high-dose cohort (Supplementary Table 10 and Supplementary Figure 2).

A third exploratory endpoint was change from baseline in HFMSE for patients in the younger group who continued in the study past 24 months of age and had at least 6 months of HFMSE data. Pre-treatment HFMSE was not assessed for patients at time of dosing in this age group because HFMSE is not valid for patients before 24 months of age. Therefore, HFMSE scoring began when patients reached 24 months of age. The median (range) change from the baseline (defined as the first assessment when patients reached 24 months of age) to 6 months post-baseline was 2.0 (–2–6) in the low-dose cohort (*n* = 2), 5.5 (1–14) in the medium-dose cohort (*n* = 6), and 5.0 (4–6) in the high-dose cohort (*n* = 2).

## DISCUSSION

STRONG is the first safety and efficacy study investigating intrathecal delivery of onasemnogene abeparvovec for SMA, demonstrating that it is safe and well-tolerated. The 32 children in this study range from very weak to stronger nonambulatory children (i.e., baseline HFMSE score of 3–32 in older patients from the medium-dose cohort), representing the broad range of functionality observed for patients with three copies of *SMN2*. Patients in the older group treated with the medium dose (1.2×10^14^ vg) demonstrated statistically significant and clinically meaningful changes in HFMSE scores compared with the primary PNCR population, with a median change from baseline that was approximately twice the three-point difference accepted as the minimal clinically meaningful change [25, 29]. Improvements were also observed in Bayley-III gross and fine motor subtest scores for the older group treated with the medium dose, although none of the patients achieved the Bayley-III motor milestone of independent walking. Although patients in the younger group did not achieve the primary or the secondary endpoints (standing independently and walking independently, respectively), improvements in Bayley-III gross and fine motor subtest total scores were observed. Because no natural history data exist for the Bayley-III for children with SMA type 2, it is not possible to know how much of these improvements are attributable to developmental maturation or drug response.

Although STRONG was not designed to compare the frequency and severity of AEs between intravenous and intrathecal administration, the safety profile of intrathecal onasemnogene abeparvovec was consistent with the cumulative experience with intravenous treatment for patients with SMA. Overall, a single administration of intrathecal onasemnogene abeparvovec was well-tolerated. Transaminase elevations (ALT/AST >3×ULN), without elevation of bilirubin, were reported in only one patient. One patient had an isolated confirmed low platelet value (<75×10^9^/mL) that resolved spontaneously with no intervention. No patients had AEs indicative of cardiac toxicity (myocardial inflammation or thrombus), and no patients had AEs of TMA. The absence of clinically observable sensory abnormalities suggestive of ganglionitis is also important given that studies in NHPs reported DRG findings following intrathecal administration [28]. This finding was not observed before the initiation of the study; therefore, serial electrophysiologic or focused clinical sensory evaluations were not performed.

Because onasemnogene abeparvovec (medium dose) at 1.2×10^14^ vg was safe and well-tolerated with demonstrated efficacy in STRONG, it was chosen for use in STEER (NCT05089656), an ongoing, randomized, sham-controlled, double-blind Phase III study to evaluate the efficacy, safety, and tolerability of intrathecal onasemnogene abeparvovec in treatment-naïve patients with SMA type 2 aged ≥2 to <18 years.

The primary efficacy endpoints for STRONG were selected because they are age- and developmentally appropriate, and reference data exist for comparison (e.g., HFMSE from PNCR [26, 31]). The HFMSE was designed specifically for children with SMA types 2 or 3 aged ≥24 months and is a key outcome measure in SMA clinical trials because the individual items and the detected changes have clear content validity and clinical meaningfulness for patients and their caregivers [30]. In STRONG, both age groups demonstrated HFMSE gains not observed for PNCR patients with three copies of *SMN2* over a 12-month period (91.7% of older patients in the medium-dose cohort achieved a three-point or greater increase in HFMSE, compared with 13.3% in the primary PNCR population) [30, 31]. These gains also contrast with a recent natural history study that included 267 SMA type 2 patients, in which 27% of patients younger than 5 years of age had a two-point gain after 12 months [32]. Important motor gains were observed across a broad range of severities, which included very weak children.

Both stabilization of function and increase in motor achievement are meaningful for individuals with SMA when compared with the natural history of progressive functional decline. SMA type 2 patients lose approximately two HFMSE points per year between the ages of 5 and 13 years [6, 33]. Two HFMSE points can represent an entire skill on this scale. In a study of 73 patients with SMA type 2, none of the patients who were 14 years of age had HFMSE scores >10, reflecting extreme weakness with the loss of abilities such as rolling, the progression of scoliosis more than 50°, and the worsening of contractures [33]. Moreover, a one-point increase in HFMSE may be considered a meaningful change to caregivers of nonambulatory patients with SMA type 2 [34].

One potential limitation of the study is the relatively short post-dose observation period (12 months for the low- and medium-dose cohorts, and 15 months for the high-dose cohort), as well as the small size for the high-dose cohort (*n* = 4). Although the 12-month period is sufficient for a clinical trial to observe improvements in both HFMSE and Bayley-III scores, it may not be adequate to evaluate the full impact of treatment on the natural history of the disease (e.g., future achievement of motor milestones, including standing alone and walking alone). The relatively short duration of this study also precludes any assessment of potential long-term toxicities associated with intrathecal onasemnogene abeparvovec administration or the potential for diminished efficacy over time. Gene-transfer therapies are by nature nonreversible and therefore necessitate systematic long-term follow-up, and all patients have been invited to participate in a separate long-term follow-up study. STRONG included symptomatic patients, who represented the spectrum from weaker to stronger “sitters.” Those at the weaker end of baseline often exhibited orthopedic, pulmonary, or other complications of weakness that may progress after onasemnogene abeparvovec therapy, even after the progressive motor neuronopathy of SMA has been tempered. Limitations in lower extremity improvement may also be because of early loss of motor neurons in lumbar and sacral regions [35], thus limiting the ability to respond to gene therapy and further emphasizing the importance of early treatment. While NHPs administered intrathecal sc-AAV9-CB-GFP demonstrated predominant systemic biodistribution [13], systemic manifestations resulting from peripheral biodistribution of onasemnogene abeparvovec were not directly evaluated in STRONG beyond the absence of AEs. Further studies are necessary to examine transduction of non-CNS tissue as well as biodistribution following intrathecal injection. Finally, biomarker studies that might enable improved short- and long-term assessment of efficacy and toxicity were not performed in this study.

## CONCLUSIONS

Intrathecal onasemnogene abeparvovec was safe and well-tolerated. Transaminase elevations (ALT/AST >3× ULN) without elevation of bilirubin were reported in only one patient. One patient had a confirmed low platelet value <75×10^9^/L that resolved spontaneously without intervention. No patients had AEs indicative of cardiac toxicity (myocardial inflammation or thrombus), TMA, or clinical manifestations indicative of sensory ganglionopathy. Intrathecal onasemnogene abeparvovec also demonstrated efficacy for SMA patients 2–5 years of age treated with the medium dose (1.2×10^14^ vg), as observed with increases in HFMSE scores from baseline that contrast with natural history, although the primary efficacy endpoint in patients 6–24 months of age was not met within the post-dose observation period of the STRONG study.

## Supplementary Material

Supplementary Material
