# Supplementary material for: Intrathecal Onasemnogene Abeparvovec for Sitting, Nonambulatory Patients with Spinal Muscular Atrophy: Phase I Ascending-Dose Study (STRONG)
Source: J Neuromuscul Dis. 2023 May 2;10(3):389–404. doi: 10.3233/JND-221560 (PMC10200150; doi:10.3233/JND-221560)
Supplement: Supplementary Material [file jnd-10-jnd221560-s001.docx]

**SUPPLEMENTARY MATERIAL**

**STRONG STUDY GROUP**

**Washington University in St. Louis**

Pallavi Anand; Natalie Goedeker

**Boston Children’s Hospital**

Abigail Druffner; Amanda Engelbrekt; Partha Ghosh; Robert Graham; Elizabeth Maczek; Amy Pasternak

**Children’s Hospital of Philadelphia**

Diane Barcoski; Allison Cullen; Jean Flickinger; Khrystine Ford; Allan Glanzman; Gunda Simpkins; Joshua Zigmont

**Nemours Children’s Hospital**

Debbie Berry; Matt Civitello; Jorge Collado; Julie Duke; Craig Johnson; Coralis Mercado Gonzalez; Aledie Navas Nazario; Fabiola Weber Guzman; Jen Wilson

**David Geffen School of Medicine at UCLA**

Clara H. Sam; Christy Skura; Loretta Staudt

**Stanford University Neurosciences Health Center**

Steven Chinn; Sally Dunaway Young; Tina Duong; Richard Gee; Shellie Josephs; Carolyn McLauglin; Jacinda Sampson; Monica Sangco

**Inclusion Criteria**

Characteristics of spinal muscular atrophy (SMA) required for enrollment in STRONG included: (1) genetic diagnosis of SMA with absence of *survival motor neuron 1* (*SMN1*) exon 7 and three copies of *SMN2* without the *SMN2* gene-modifier mutation [c.859G > C]; (2) ≥6 months of age and up to 60 months (1800 days) of age at time of dosing; (3) able to sit unassisted for 10 or more seconds (WHO-MGRS criteria) but unable to stand or walk independently; and (4) manifested clinical signs and symptoms consistent with SMA prior to 12 months of age. Other inclusion criteria include patients able to meet age-appropriate institutional criteria for use of anesthesia and sedation, as determined necessary by the investigator, up to date on childhood vaccines, and palivizumab prophylaxis (also known as Synagis^®^) to prevent respiratory syncytial virus was also recommended in accordance with the American Academy of Pediatrics Guideline [1], and parent(s)/legal guardian were willing and able to complete the informed consent process.

**Exclusion Criteria**

Patients were excluded from enrollment for any of the following criteria: (1) current or historic ability to stand or walk independently; (2) contraindications for spinal tap procedure or administration of intrathecal therapy (e.g., spina bifida, meningitis, impairment, or clotting abnormalities, or obstructive spinal hardware preventing effective access to cerebrospinal fluid [CSF] space) or presence of an implanted shunt for the drainage of CSF or an implanted central nervous system catheter; (3) severe contractures as determined by designated physical therapist(s) at screening that interfere with either the ability to attain/demonstrate functional measures (e.g., standing, walking) or interferes with ability to receive intrathecal dosing; (4) severe scoliosis (defined as ≥50° Cobb angle) evident on x-ray examination; (5) previous, planned, or expected scoliosis repair surgery/procedure within 1 year of dose administration; (6) use of invasive ventilatory support (tracheostomy with positive pressure) or pulse oximetry <95% saturation at screening while the patient is awake, or for high altitudes >1000 m, oxygen saturation <92% while the patient is awake (pulse oximetry saturation must not decrease ≥4 percentage points between screening and greatest value on day of dosing); (7) use or requirement of noninvasive ventilatory support for 12 or more hours daily in the 2 weeks prior to dosing; (8) medical necessity for a gastric feeding tube, in which the majority of feedings are given by non-oral methods (i.e., nasogastric tube or nasojejunal tube) or patients whose weight-for-age falls below the 3rd percentile based on WHO Child Growth Standards—placement of a permanent gastrostomy prior to screening is not an exclusion; (9) active viral infection (includes human immunodeficiency virus or serology positive for hepatitis B or C, or Zika virus); (10) serious non–respiratory tract illness requiring systemic treatment and/or hospitalization within 2 weeks prior to study entry; (11) respiratory infection requiring medical attention, medical intervention, or increase in supportive care of any manner within 4 weeks prior to study entry; (12) severe non–pulmonary/respiratory tract infection (e.g., pyelonephritis, or meningitis) within 4 weeks before study dosing or concomitant illness that in the opinion of the principal investigator creates unnecessary risks for gene transfer such as major renal or hepatic impairment, known seizure disorder, diabetes mellitus, idiopathic hypocalciuria or symptomatic cardiomyopathy; (13) history of bacterial meningitis or brain or spinal cord disease, including tumors, or abnormalities by magnetic resonance imaging or computed tomography that would interfere with the lumbar puncture procedures or CSF circulation; (14) known allergy or hypersensitivity to prednisolone or other glucocorticosteroids or their excipients; (15) known allergy or hypersensitivity to iodine or iodine-containing products; (16) concomitant use of any of the following: drugs for treatment of myopathy or neuropathy, agents used to treat diabetes mellitus, or ongoing immunosuppressive therapy, plasmapheresis, immunomodulators such as adalimumab, or immunosuppressive therapy within 3 months of study dosing (e.g., corticosteroids, cyclosporine, tacrolimus, methotrexate, cyclophosphamide, intravenous immunoglobulin, rituximab); (17) inability to withhold use of laxatives or diuretics in the 24 hours prior to dose administration; (18) anti–adeno-associated virus serotype 9 (AAV9) antibody titers >1:50 as determined by enzyme-linked immunosorbent assay binding immunoassay; should a potential patient demonstrate anti-AAV9 antibody titer >1:50, he or she may receive retesting within 30 days of the screening period and will be eligible to participate if the anti-AAV9 antibody titer upon retesting is ≤1:50; (19) clinically significant abnormal laboratory values (gamma-glutamyl transferase, alanine aminotransferase, and aspartate aminotransferase, or total bilirubin >2× upper limit of normal, creatinine ≥1.0 mg/dL, hemoglobin <8 or >18 g/dL, white blood cell >20,000 per cmm) prior to gene replacement therapy; patients with an elevated bilirubin concentration that is unequivocally the result of neonatal jaundice shall not be excluded; (20) participation in recent SMA treatment clinical trial or receipt of an investigational or approved compound product or therapy received with the intention to treat SMA (e.g., valproic acid, nusinersen) at any time prior to screening for this study; oral beta-agonists must be discontinued 30 days prior to dosing but inhaled albuterol specifically prescribed for the purposes of respiratory (bronchodilator) management was acceptable and not a contraindication at any time prior to screening for this study; (21) expectation of major surgical procedures during the 1-year study assessment period (e.g., spinal surgery or tracheostomy); (22) inability or unwillingness to comply with study procedures or inability to travel for repeat visits; (23) unwillingness to keep study results/observations confidential or to refrain from posting confidential study results/observations on social media sites; or (24) refusal to sign consent form by parent(s)/legal guardian(s).

**Identification of Adverse Events of Special Interest**

The following specific treatment-emergent adverse events of special interest were primarily identified by using Standardized Medical Dictionary for Regulatory Activities (MedDRA^®^) queries (SMQ) and Customized MedDRA queries (CMQ):

- Hepatotoxicity, identified via the following SMQ: hepatic disorders (SMQ)
- Thrombocytopenia, identified via the following CMQ: transient thrombocytopenia (CMQ)
- Cardiac events, identified via the following SMQs: ischemic heart disease (SMQ), cardiomyopathy (SMQ), cardiac arrhythmias (SMQ), embolic and thrombotic events (SMQ), and myocardial infarction (SMQ)
- Thrombotic microangiopathy, identified via the following approach:
  - Criteria #1: cases with any one of the following preferred terms (PTs): thrombotic microangiopathy OR hemolytic uremic syndrome OR atypical hemolytic uremic syndrome
  - Criteria #2: cases with at least one PT from each of the following SMQs representing thrombocytopenia, hemolysis, and relevant renal events, respectively: hematopoietic thrombocytopenia (SMQ), hemolytic disorders (SMQ), acute renal failure (SMQ), or renovascular disorders (SMQ)
- Sensory abnormalities suggestive of ganglionitis, identified via the following CMQ: dorsal root ganglia (DRG) cell inflammation (CMQ)

**Supplemental Table 1. Summary of serious TEAEs by preferred term (safety population)**

| **Preferred term** | **Low dose**  **(6.0×10^13^ vg; n=3)** | **Medium dose** **(1.2×10^14^ vg; n=25)** | | **High dose** **(2.4×10^14^ vg; n=4)** | **Overall**  **(N=32)** |
| --- | --- | --- | --- | --- | --- |
|  | **Younger group,**  **n (%)** | **Younger group,**  **n (%)** | **Older group,**  **n (%)** | **Younger group,**  **n (%)** | **All ages,**  **n (%)** |
| **Any TEAE** | 1 (33.3) | 2 (15.4) | 4 (33.3) | 0 | 7 (21.9) |
| Blood alkaline phosphatase increased | 1 (33.3) | 1 (7.7) | 0 | 0 | 2 (6.3) |
| Bronchitis | 0 | 0 | 1 (8.3) | 0 | 1 (3.1) |
| Influenza | 0 | 0 | 1 (8.3) | 0 | 1 (3.1) |
| Pneumonia | 0 | 0 | 1 (8.3) | 0 | 1 (3.1) |
| Pneumonia respiratory syncytial viral | 0 | 0 | 1 (8.3) | 0 | 1 (3.1) |
| Respiratory tract infection viral | 0 | 0 | 1 (8.3) | 0 | 1 (3.1) |
| Rhinovirus infection | 0 | 0 | 1 (8.3) | 0 | 1 (3.1) |
| Alanine aminotransferase increased | 0 | 1 (7.7) | 0 | 0 | 1 (3.1) |
| Aspartate aminotransferase increased | 0 | 1 (7.7) | 0 | 0 | 1 (3.1) |
| Acute respiratory failure | 0 | 0 | 1 (8.3) | 0 | 1 (3.1) |
| Asthma | 0 | 0 | 1 (8.3) | 0 | 1 (3.1) |
| Respiratory failure | 1 (33.3) | 0 | 0 | 0 | 1 (3.1) |

TEAE, treatment-emergent adverse event; vg, vector genomes.

Note: TEAEs are classified by preferred term using MedDRA^®^, Version 23.0.

Younger group, 6 to <24 months of age at dosing; older group, 24 to <60 months of age at dosing.

**Supplemental Table 2. Summary of study drug-related TEAEs by preferred term (safety population)**

| **Preferred term** | **Low dose (6.0×10^13^ vg; n=3)** | **Medium dose** **(1.2×10^14^ vg; n=25)** | | **High dose** **(2.4×10^14^ vg; n=4)** | **Overall**  **(N=32)** |
| --- | --- | --- | --- | --- | --- |
|  | **Younger group,  n (%)** | **Younger group,**  **n (%)** | **Older group,**  **n (%)** | **Younger group,**  **n (%)** | **All ages,**  **n (%)** |
| **Any TEAE** | 0 | 7 (53.8) | 4 (33.3) | 1 (25.0) | 12 (37.5) |
| Hypertension | 0 | 3 (23.1) | 0 | 0 | 3 (9.4) |
| Aspartate aminotransferase increased | 0 | 1 (7.7) | 1 (8.3) | 0 | 2 (6.3) |
| Lymphadenopathy | 0 | 1 (7.7) | 1 (8.3) | 0 | 2 (6.3) |
| Pyrexia | 0 | 1 (7.7) | 1 (8.3) | 0 | 2 (6.3) |
| Vomiting | 0 | 1 (7.7) | 0 | 1 (25.0) | 2 (6.3) |
| Activated partial thromboplastin time prolonged | 0 | 1 (7.7) | 0 | 0 | 1 (3.1) |
| Alanine aminotransferase increased | 0 | 1 (7.7) | 0 | 0 | 1 (3.1) |
| Blood creatine phosphokinase MB increased | 0 | 0 | 1 (8.3) | 0 | 1 (3.1) |
| Cardiac murmur | 0 | 1 (7.7) | 0 | 0 | 1 (3.1) |
| Hair growth abnormal | 0 | 0 | 1 (8.3) | 0 | 1 (3.1) |
| Hepatomegaly | 0 | 1 (7.7) | 0 | 0 | 1 (3.1) |
| Pericardial effusion | 0 | 1 (7.7) | 0 | 0 | 1 (3.1) |
| Sinus tachycardia | 0 | 1 (7.7) | 0 | 0 | 1 (3.1) |

MB, muscle/brain; TEAE, treatment-emergent adverse event; vg, vector genomes.

Younger group, 6 to <24 months of age at dosing; older group, 24 to <60 months of age at dosing.

Note: TEAEs are classified by preferred term using MedDRA^®^, Version 23.0.

Study drug-related adverse events are events that are considered possibly, probably, or definitely related to onasemnogene abeparvovec by the investigator.

Note: One patient had a serious TEAE that was considered related to treatment.

**Supplemental Table 3. TEAEs of special interest: hepatotoxicity (enrolled population)**

|  | **Age (months)** | **TEAE (preferred term)** | **Onset (study day)/**  **resolution (study day)/**  **AE duration (days)** | **Serious (Y/N)/ severity** | **Outcome** | **Causality per investigator** | **Prednisolone or**  **prednisolone equivalent**  **daily dosage** |
| --- | --- | --- | --- | --- | --- | --- | --- |
| **Low dose** | | | | | | | |
| Patient 1 | 12.5 | Blood alkaline phosphatase increased | 182/217/36 | Y / Grade 3 | Recovered/ resolved | Unrelated | 1 mg/kg Days −1 to 30  0.5 mg/kg Days 31 to 38  0.25 mg/kg Days 39 to 46  Total days=47 |
| **Medium dose** | | | | | | | |
| Patient 6 | 11.9 | Blood alkaline phosphatase increased | 364 | Y / Grade 3 | Recovered/  resolved with sequelae | Unrelated | 1 mg/kg Days −1 to 30  0.5 mg/kg Days 31 to 37  0.25 mg/kg Days 38 to 44  Total days=45 |
| Patient 11 | 7.0 | Blood alkaline phosphatase increased | 127/182/56 | N / Grade 3 | Recovered/ resolved | Unrelated | 1 mg/kg Days −1 to 35  0.5 mg/kg Days 36 to 56  0.25 mg/kg Days 57 to 70  Total days=71 |
| Patient 13 | 12.9 | Blood alkaline phosphatase increased | 84/116/33 | N / Grade 3 | Recovered/ resolved | Unrelated | 1 mg/kg Days −1 to 29  0.5 mg/kg Days 30 to 43  0.25 mg/kg Days 44 to 57  Total days=58 |
| Patient 14 |  | Hepatomegaly | 16/30/15 | N / Grade 1 | Recovered/ resolved | Probably related | 1 mg/kg Days −1 to 32  0.5 mg/kg Days 33 to 50  0.25 mg/kg Days 51 to 61  For ALT/AST elevations^a^:  12.9 mg Days 61 to 98  9 mg Days 99 to 105  4.5 mg Days 106 to 112  2.1 mg Days 113 to 120  Total days=121 |
|  |  | ALT increased | 59/93/35 | Y / Grade 3 | Recovered/ resolved | Probably related |  |
|  | 14.3 | AST increased | 59/71/13 | Y / Grade 2 | Recovered/ resolved | Probably related |  |
| Patient 20 | 54.5 | AST increased | 63/360/298 | N / Grade 1 | Recovered/ resolved | Probably related | 1 mg/kg Days −1 to 31  0.5 mg/kg Days 32 to 42  0.25 mg/kg Days 43 to 57  Total days=58 |
| **High dose** | | | | | | | |
| Patient 30 | 17.3 | Blood alkaline phosphatase increased | 218/275/58 | N / Grade 2 | Recovered/  resolved | Unrelated | 1 mg/kg Days −1 to 35  0.5 mg/kg Days 36 to 50  0.25 mg/kg Days 51 to 64  Total days=65 |

AE, adverse event; ALT, alanine aminotransferase; AST, aspartate aminotransferase; TEAE, treatment-emergent adverse event.

^a^Recorded as concomitant medication.

Note: TEAEs are classified by preferred term using MedDRA^®^, Version 23.0.

**Supplemental Table 4. TEAEs of special interest: thrombocytopenia (enrolled population)**

|  | **Age (months)** | **TEAE (preferred term)** | **Onset (study day)/**  **resolution (study day)/**  **AE duration (days)** | **Outcome** | **Causality per investigator** | **Comment** |
| --- | --- | --- | --- | --- | --- | --- |
| **Low dose** | | | | | | |
| Patient 3 | 18.9 | Infusion site bruising | 3/8/6 | Recovered/resolved | Unrelated | Platelets Day 2 = 57×10^9^/L, but returned to normal on the following test on Day 8 |
| **Medium dose** | | | | | | |
| Patient 4 | 20.4 | Contusion | 1/15/15 | Recovered/resolved | Unrelated | No associated decrease in  platelets |
| Patient 6 | 11.9 | Contusion | 21/35/15 | Recovered/resolved | Unrelated | No associated decrease in  platelets |
| Patient 8 | 20.3 | Hematochezia | 14/28/15 | Recovered/resolved | Unrelated | No associated decrease in  platelets |
| Patient 14 | 14.3 | Activated partial thromboplastin time prolonged | 93 | Not recovered/not  resolved | Probably related | No associated decrease in  platelets |

AE, adverse event; TEAE, treatment-emergent adverse event.

Note: TEAEs are classified by preferred term using MedDRA^®^, Version 23.0.

**Supplemental Table 5. TEAEs of special interest: cardiac events (enrolled population)**

|  | **Age (months)** | **TEAE (preferred term)** | **Onset (study day)/**  **resolution (study day)/**  **AE duration (days)** | **Outcome** | **Causality per investigator** |
| --- | --- | --- | --- | --- | --- |
| **Medium dose** | | | | | |
| Patient 4 | 20.4 | Blood pressure diastolic increased | 8/15/8 | Recovered/resolved | Unrelated |
| Patient 6 | 11.9 | Sinus tachycardia | 91/364/274 | Recovered/resolved | Possibly related |
| Patient 7 | 23.1 | Electrocardiogram  QT prolonged | 175/175/1 | Recovered/resolved | Unrelated |
| Patient 8 | 20.3 | Tachycardia | 265/301/37 | Recovered/resolved | Unrelated |
| Patient 10 | 19.8 | Tachycardia | 2/2/1 | Recovered/resolved | Unrelated |
|  |  | Tachycardia | 302 | Not recovered/not  resolved | Unrelated |
| Patient 14 | 14.3 | Hepatomegaly | 16/30/15 | Recovered/resolved | Probably related |
| Patient 20 | 54.5 | Blood creatine phosphokinase MB increased | 63/360/298 | Recovered/resolved | Possibly related |
|  |  | Tachycardia | 360 | Not recovered/not  resolved | Unrelated |
| Patient 21 | 35.6 | Sinus tachycardia | 1/3/3 | Recovered/resolved | Unrelated |
| Patient 26 | 27.3 | Cardiomegaly | 271/276/6 | Recovered/resolved | Unrelated |
|  |  | Tachycardia | 276/276/1 | Recovered/resolved | Unrelated |

AE, adverse event; TEAE, treatment-emergent adverse event.

Note: TEAEs are classified by preferred term using MedDRA^®^, Version 23.0.

**Supplemental Table 6. MMRM analysis of change from baseline in HFMSE scores at
12 months — patients in the older group (ITT population)**

|  | **Primary PNCR population**  **(n=15)^a^** | **Intrathecal onasemnogene abeparvovec**  **Medium dose**  **(1.2×10^14^ vg; n=12)** |
| --- | --- | --- |
| **Baseline** |  |  |
| n | 15 | 12 |
| Median (range) | 9.0 (0–22) | 12.0 (3–32) |
| **Month 12: Actual value** |  |  |
| N | 9 | 12 |
| Median (range) | 10.0 (0–22) | 16.5 (6–40) |
| **Month 12: Change from baseline** | | |
| N | 9 | 12 |
| MMRM^b^ | | |
| LS mean (95% CI) | 0.5 (–2.2, 3.2) | 6.0 (3.7, 8.3) |
| Difference between LS mean (95% CI) | | 5.5 (1.9, 9.0) |
| *P*-value | | 0.01 |

HFMS, Hammersmith Functional Motor Scale; HFMSE, Hammersmith Functional Motor Scale Expanded; ITT, intention-to-treat; LS, least square; MMRM, mixed model with repeated measure; PNCR, Pediatric Neuromuscular Clinical Research; vg, vector genomes.

Older group, 24 to <60 months of age at dosing.

^a^Includes a subset of 15 patients from the PNCR natural history control population who had SMA types 2 or 3, three copies of *SMN2*, symptom onset before 12 months of age, diagnosis before 24 months of age, were unable to stand or walk at enrollment (baseline visit), received an HFMSE evaluation between 24 and 60 months of age (“baseline”), and had a follow-up evaluation (HFMS of HFMSE performed between 12 and 14 months following that baseline evaluation.

^b^MMRM included the change from baseline as the dependent variable, fixed effect of cohort (intrathecal onasemnogene abeparvovec and PNCR), visit, covariates of baseline HFMSE and age at baseline, and interactions of cohort by age at baseline, baseline HFMSE by visit, baseline HFMSE by cohort, and cohort by visit. A compound symmetry covariance structure is assumed initially to model the within-patient errors.

**Supplemental Table 7. Percentage of patients in the older group achieving ≥3-point increase in HFMSE at any post-baseline visit up to 12 months (ITT population)**

|  | **Statistics** | **Primary**  **PNCR population^a^**  **(n=15)** | **Intrathecal onasemnogene abeparvovec**  **Medium dose**  **(1.2×10^14^ vg; n=12)** |
| --- | --- | --- | --- |
| Percentage of patients achieving ≥3-point increase in HFMSE, n (%) | Yes | 2 (13.3) | 11 (91.7) |
|  | No | 13 (86.7) | 1 (8.3) |
| Percentage difference test | Difference in percentage vs. PNCR (95% CI) | | 78.3 (42.5, 95.0) |
|  | *P*-value (Fisher’s exact test) | | <0.01 |

HFMS, Hammersmith Functional Motor Scale; HFMSE, Hammersmith Functional Motor Scale-Expanded; ITT, intention-to-treat; PNCR, Pediatric Neuromuscular Clinical Research; vg, vector genomes.

^a^Includes a subset of 15 patients from the PNCR natural history control population who had SMA types 2 or 3, three copies of *SMN2*, symptom onset before 12 months of age, diagnosis before 24 months of age, were unable to stand or walk at enrollment (baseline visit), received an HFMSE evaluation between 24 and 60 months of age (“baseline”), and had a follow-up evaluation (HFMS of HFMSE performed between 12 and 14 months following that baseline evaluation.

Older group, 24 to <60 months of age at dosing.

**Supplemental Table 8. Percentage of patients in the younger group achieving the ability to walk without assistance at any post-baseline visit up to 12 months (ITT population)**

|  | **PNCR natural history control population^a^** | **Onasemnogene abeparvovec** | | |
| --- | --- | --- | --- | --- |
|  | **(n=51)** | **Low dose**  **(6×10^13^ vg;  n=3)** | **Medium dose**  **(1.2×10^14^ vg; n=13)** | **High dose**  **(2.4×10^14^ vg; n=4)** |
| **Percentage of patients achieving the ability to walk without assistance, n (%)** | | | | |
| Yes | 5 (9.8) | 0 | 1 (7.7) | 0 |
| No | 46 (90.2) | 3 (100.0) | 12 (92.3) | 4 (100) |
| **Percentage difference test^b^** | | | | |
| Difference in percentage vs. PNCR (95% CI) | |  | –2.1 (–17.2, 27.0) |  |
| *P*-value (Fisher’s exact test) | |  | >0.999 |  |

ITT, intention-to-treat; PNCR, Pediatric Neuromuscular Clinical Research; vg, vector genomes.

Younger group, 6 to <24 months of age at dosing.

^a^Includes all patients enrolled in the PNCR study who met the criteria of having SMA types 2 or 3, three copies of *SMN2*, symptom onset before 12 months of age, and at least one visit at or before 36 months of age.

^b^The Fisher exact test was only performed for the medium-dose cohort.

**Supplemental Table 9.** **Bayley-III motor milestones achieved beyond independent sitting by patients (enrolled population)**

| **Patient** | **Age at dosing (months)** | **Bayley-III gross motor milestones** | **Age at achievement (months)^a^** |
| --- | --- | --- | --- |
| **Younger group** | | | |
| **Low dose** | | | |
| Patient 2 | 20.2 | Pulls to stand, #35  Crawls, #34  Stands alone, #40 | 25.4  26.5  31.3 |
| Patient 3 | 18.9 | Crawls, #34 | 19.8 |
| **Medium dose** | | | |
| Patient 6 | 11.9 | Crawls, #34  Stands with assistance, #33 | 24.0  24.0 |
| Patient 9 | 14.7 | Rolls from back to sides, #20 | 16.6 |
| Patient 11 | 7.0 | Crawls, #34  Pulls to stand, #35  Stands with assistance, #33  Stands alone, #40  Walks with assistance, #37  Walks alone, #43 | 7.9  7.9  7.9  9.8  11.2  11.2 |
| Patient 14 | 14.3 | Rolls from back to sides, #20 | 17.4 |
| Patient 15 | 17.7 | Rolls from back to sides, #20  Stands with assistance, #33  Pulls to stand, #35  Walks with assistance, #37 | 19.5  19.5  28.5  28.5 |
| Patient 16 | 15.3 | Rolls from back to sides, #20 | 17.0 |
| **High dose** | | | |
| Patient 29 | 17.4 | Rolls from back to sides, #20 | 18.4 |
| Patient 31 | 23.2 | Pulls to stand, #35 | 41.3 |
| Patient 32 | 9.5 | Stands with assistance, #33 | 24.7 |
| **Older group** | | | |
| **Medium dose** | | | |
| Patient 17 | 30.7 | Stands with assistance, #33 | 34.4 |
| Patient 19 | 50.3 | Stands with assistance, #33  Walks with assistance, #37 | 53.3  55.1 |
| Patient 26 | 27.3 | Rolls from back to sides, #20 | 28.3 |

Bayley-III, Bayley Scales of Infant and Toddler Development, Version 3.

Younger group, 6 to <24 months of age at dosing; older group, 24 to <60 months of age at dosing.

^a^Ages represent the age motor milestones were documented at a study visit and not the date when actually first achieved.

Note: All other patients had no measurable Bayley-III motor milestones.

**Supplemental Table 10. Maximum change from baseline at any post-baseline visit up to 12 months in the Bayley-III gross and fine motor scores for patients in the younger group (ITT population)**

| **Category visit**  **statistics** | **Onasemnogene abeparvovec** | | |
| --- | --- | --- | --- |
|  | **Low dose**  **(6×10^13^ vg; n=3)** | **Medium dose**  **(1.2×10^14^ vg; n=13)** | **High dose**  **(2.4×10^14^ vg; n=4)** |
| **Gross motor** | | | |
| **Baseline** | | | |
| Median (range) | 28.0 (17–34) | 20.0 (14–30) | 22.0 (18–32) |
| **Post-baseline value for the visit with maximum change from baseline observed value** | | | |
| Median (range) | 33.0 (24–39) | 24.0 (18–55) | 28.0 (24–34) |
| **Change from baseline** | | |  |
| Median (range) | 5.0 (5–7) | 5.0 (1–25) | 4.0 (2–10) |
| **Fine motor** | | | |
| **Baseline** | | | |
| Median (range) | 33.0 (28–33) | 31.0 (22–38) | 32.5 (22–43) |
| **Post-baseline value for the visit with maximum change from baseline observed value** | | | |
| Median (range) | 46.0 (42–52) | 45.0 (37–50) | 43.0 (41–51) |
| **Change from baseline** | | | |
| Median (range) | 18.0 (9–19) | 12.0 (7–19) | 10.5 (8–19) |

ITT, intention-to-treat; vg, vector genomes.

Younger group, 6 to <24 months of age at dosing.

**Supplemental Table 11. Maximum change from baseline up to 12 months in Bayley-III gross and fine motor scores for patients in the older group (ITT population)**

| **Category visit statistics** | **Onasemnogene abeparvovec**  **Medium dose**  **(1.2×10^14^ vg; n=12)** |
| --- | --- |
| **Gross motor** | |
| **Baseline** | |
| Median (range) | 20.0 (16–35) |
| **Post-baseline value for the visit with maximum change from baseline observed value** | |
| Median (range) | 25.0 (18–39) |
| **Change from baseline** | |
| Median (range) | 3.0 (1–12) |
| **Fine motor** | |
| **Baseline** | |
| Median (range) | 47.0 (32–60) |
| **Post-baseline value for the visit with maximum change from baseline observed value** | |
| Median (range) | 57.5 (49–65) |
| **Change from baseline** | |
| Median (range) | 10.5 (1–23) |

ITT, intention-to-treat; vg, vector genomes.

Older group, 24 to <60 months of age at dosing.

**Supplemental Figure 1. Study design**


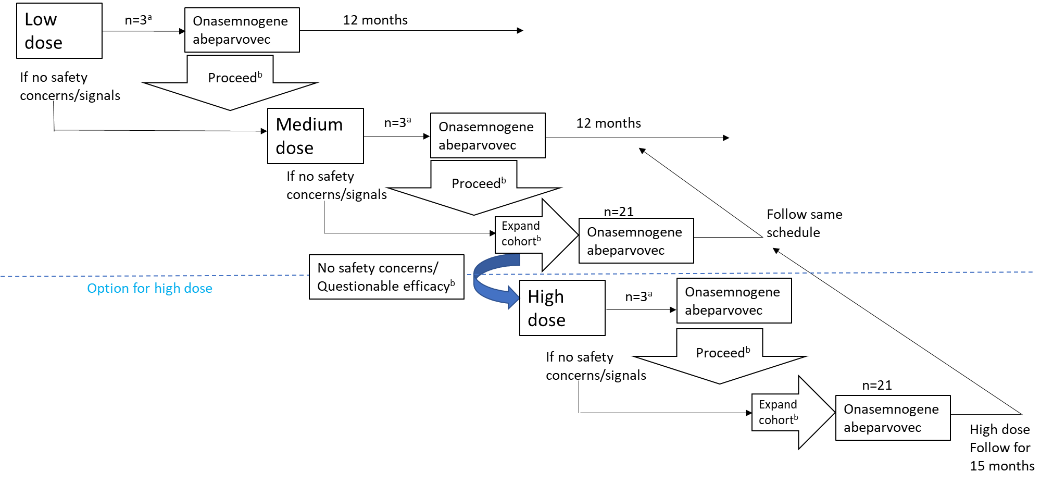


DMC, Data Monitoring Committee.

^a^Four weeks between dosing first three patients within each cohort to allow safety monitoring.

^b^Provided no dose-related toxicities and DMC determines acceptable to escalate/expand during quarterly meeting.

**Supplemental Figure 2. Change from baseline in gross motor score of Bayley scale at each post-baseline visit up to 12 months in patients in the younger group (ITT population)**

1. **Low dose, n=3**

**
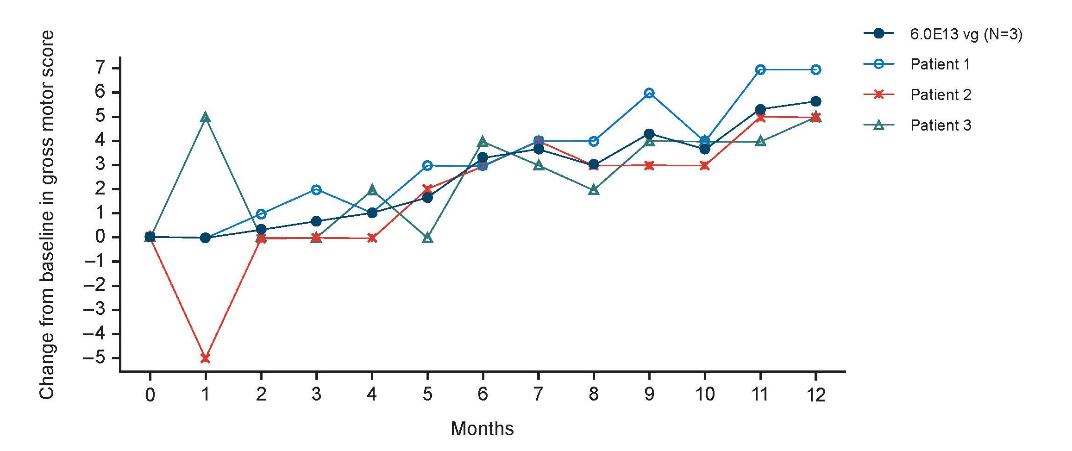
**

1. **Medium dose, n=13**

**
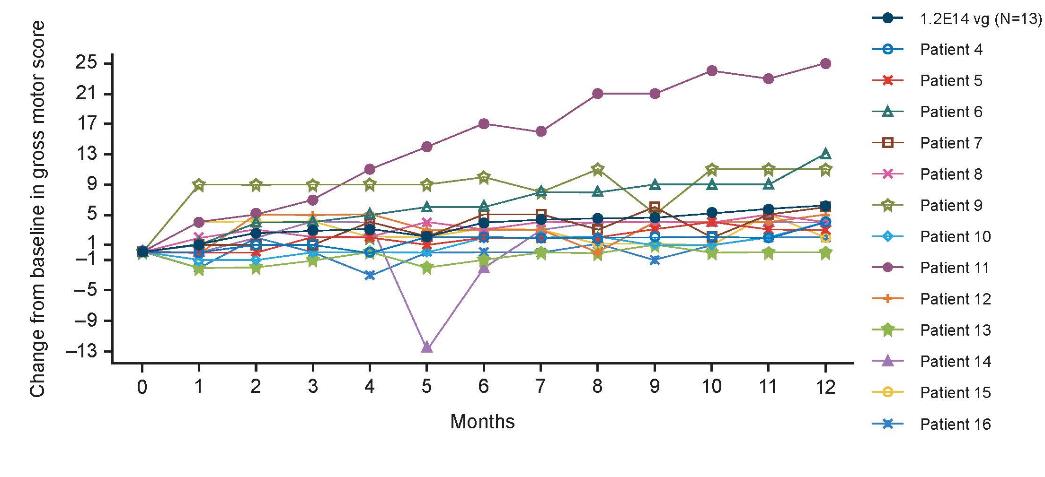
**

1. **High dose, n=4**

**
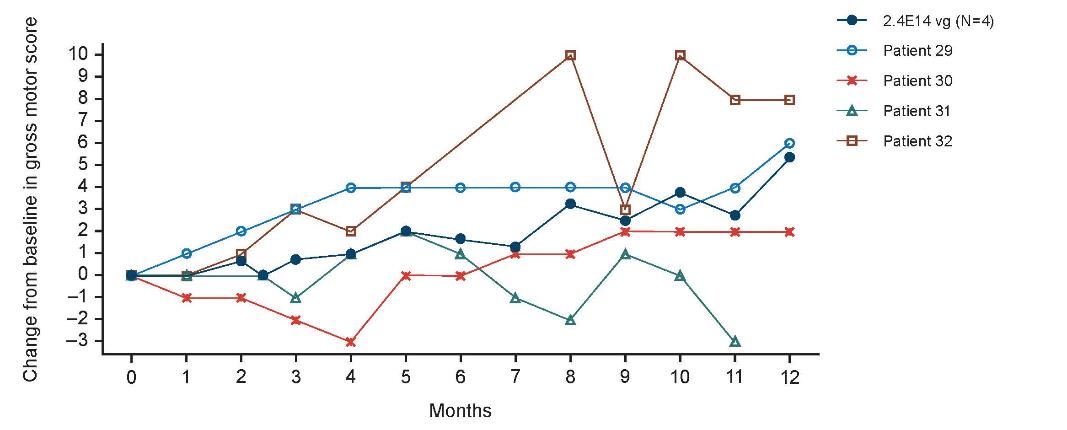
**

ITT, intention-to-treat; vg, vector genomes.

**Supplemental Figure 3. Change from baseline in gross motor score of Bayley scale at each post-baseline visit up to 12 months for patients in the older group (ITT population)**

**
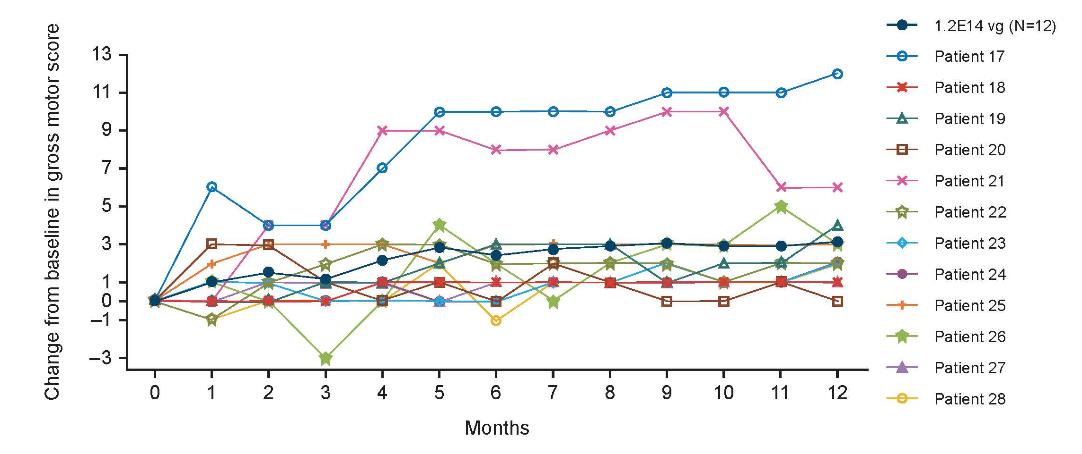
**

ITT, intention-to-treat; vg, vector genomes.

**Reference**

1. Committee on Infectious Diseases. From the American Academy of Pediatrics: Policy statements--Modified recommendations for use of palivizumab for prevention of respiratory syncytial virus infections. Pediatrics. 2009;124(6):1694–701.
